# Sequestering of damage-associated molecular patterns (DAMPs): a possible mechanism affecting the immune-stimulating properties of aluminium adjuvants

**DOI:** 10.1007/s12026-017-8972-5

**Published:** 2017-11-27

**Authors:** Andreas Svensson, Tove Sandberg, Peter Siesjö, Håkan Eriksson

**Affiliations:** 1 Lund Stem Cell Center, BMC B10, Lund University, Lund, Sweden; 2 Department of Biomedical Science, Faculty of Health and Society, Malmö University, SE-205 06 Malmö, Sweden; 3 Glioma Immunotherapy Group, Neurosurgery, Department of Clinical Sciences, BMC D14, Lund University, SE-221 84 Lund, Sweden

**Keywords:** Alarmins, Aluminium-based adjuvant, Damp, Lumogallion, Sterile inflammation

## Abstract

Aluminium-based adjuvants (ABAs) have been used in human and veterinary vaccines for decades, and for a long time, the adjuvant properties were believed to be mediated by an antigen depot at the injection site, prolonging antigen exposure to the immune system. The depot hypothesis is today more or less abandoned, and instead replaced by the assumption that ABAs induce an inflammation at the injection site. Induction of an inflammatory response is consistent with immune activation initiated by recognition of molecular patterns associated with danger or damage (DAMPs), and the latter are derived from endogenous molecules that normally reside intracellularly. When extracellularly expressed, because of damage, stress or cell death, a sterile inflammation is induced. In this paper, we report the induction of DAMP release by viable cells after phagocytosis of aluminium-based adjuvants. Two of the most commonly used ABAs in pharmaceutical vaccine formulations, aluminium oxyhydroxide and aluminium hydroxyphosphate, induced a vigorous extracellular expression of the two DAMP molecules calreticulin and HMGB1. Concomitantly, extracellular adjuvant particles adsorbed the DAMP molecules released by the cells whereas IL-1β, a previously reported inflammatory mediator induced by ABAs, was not absorbed by the adjuvants. Induction of extracellular expression of the two DAMP molecules was more prominent using aluminium hydroxyphosphate compared to aluminium oxyhydroxide, whereas the extracellular adsorption of the DAMP molecules was more pronounced with the latter. Furthermore, it is hypothesised how induction of DAMP expression by ABAs and their concomitant adsorption by extracellular adjuvants may affect the inflammatory properties of ABAs.

## Introduction

Aluminium-based adjuvants, ABAs, have been used in pharmaceutical vaccine formulations for decades, and for many years, the prolonged release of antigen at the inoculation site was regarded as the mechanism of the immune-stimulating properties of ABAs [1]. However, it has also been proposed that ABAs induce inflammation, activating the innate immune system and thereby an adaptive response [2–4]. Several reports have verified that ABAs trigger an inflammatory response, and infiltration of immune cells at the inoculation site initiates activation and maturation of innate and adaptive immune cells [5–7], with a direct effect on antigen-presenting cells [8]. The consensus has been that the immune-stimulating effects of ABAs are pleotropic and involve multiple signalling pathways.

The NLRP3-inflammasome has been reported to play an important role in the inflammatory response induced by ABAs. Activation of NLRP3-inflammasomes by ABAs initiates the cleavage and release of the pro-inflammatory cytokines IL-1β and IL-18 [2, 9, 10]. However, the NLRP3-inflammasome can also be activated by endogenous danger-associated molecular patterns, DAMPs [11]. ABA-induced release of pro-inflammatory cytokines has so far only been reported for cytokines of the IL-1 family, and in the context of ABAs, release of DAMP molecules has been claimed to emanate from injured or necrotic cells, due to endocytosis of the adjuvant [3, 4, 12].

The most common types of aluminium salts used as adjuvants are aluminium oxyhydroxide, AlO(OH), in this paper referred to as Alhydrogel, or aluminium hydroxyphosphate, Al(OH)x (PO_4_)y, here referred to as Adju-Phos. Considering the extensive use of these two ABAs, knowledge and understanding regarding the mechanisms underlying the immune response induced are surprisingly limited. In this paper, we show that viable cells release DAMP molecules upon in vitro exposure to both Alhydrogel and Adju-Phos. Release of DAMP molecules is exemplified by calreticulin and the high mobility group box 1 protein (HMGB1). Calreticulin is an intracellular homeostasis regulator of Ca^2+^ as well as chaperone controlling the quality of newly synthesised proteins in the endoplasmic reticulum [13, 14], and cells undergoing immunogenic cell death have been shown to secrete calreticulin [13]. HMGB1, on the other hand, is a non-histone chromosomal binding protein normally located in the nucleus, regulating chromosome stability [15]. Though calreticulin and HMGB1 have completely different intracellular locations, both have been shown to be secreted upon immunogenic cell death and studies have implicated a diagnostic value of both molecules in cancer therapy [13, 16, 17].

## Materials and methods

### Materials

The aluminium adjuvant preparations used in this study were Alhydrogel; AlO(OH) and Adju-Phos, Al(OH)x(PO_4_)y, purchased from Brenntag Biosector (Frederikssund, Denmark). Dealuminated zeolite Y (USY) was purchased from Tosoh Corporation, Japan.

Lumogallion (CAS 4386–25-8) was purchased from TCI Europe N.V., Antwerp, Belgium, and lipopolysaccharide (LPS, from *Escherichia coli* O111:B4) was purchased from Sigma-Aldrich, St. Louis, MO, USA.

### Cell culture

THP-1 (ATCC TIB-202) was obtained from LGC Standards, UK, and cultured in RPMI 1640 medium supplemented with 10% heat-inactivated fetal calf serum of EU grade, (Gibco, ThermoFisher Scientific) and 100 μg/ml of gentamicin (Corning Media Tech, ThermoFisher Scientific). This medium will be referred to as R10. All cells were cultured at 37 °C in a humidified atmosphere with 5% CO_2_, and the cells were maintained by sub-culturing once every third day.

### Co-culture with aluminium adjuvants and dealuminated zeolite Y

Triplicates of THP-1 cells, 0.5 × 10^6^ cells per ml, were co-cultured in 96-well plates with Alhydrogel or Adju-Phos corresponding to final aluminium concentrations ranging from 25 to 100 μg/ml in a total volume of 200 μl R10 during 1 to 16 h (over night) at 37 °C. Cells cultured in R10 in the absence of aluminium adjuvant were used as control. Specified concentrations of aluminium and incubation periods of each experiment are described in the figure legends. Cells from three to five wells of each incubation were pooled and centrifuged for 5 min at 1000×*g*. The supernatants were collected, re-centrifuged for 10 min at 13,000×*g* and then divided into aliquots and stored at − 80 °C until DAMP or cytokine content were assayed. Collected cells were re-suspended in PBS containing 0.1% (*w*/*v*) BSA and 0.1% (*w*/*v*) human IgG at 1 × 10^6^ cells per ml. The cells were sub-divided into aliquots and stained with APC-labelled anti-human calreticulin, anti-human HMGB1, anti-human IL-1β, or an APC labelled isotype control (all antibodies from R&D Systems, Minneapolis, MN, USA) by incubation for 30 min on ice. Finally, the cells were washed with PBS containing 0.1% (*w*/*v*) BSA, re-suspended in 250 μl 1% (*w*/*v*) paraformaldehyde (PFA) and analysed by flow cytometry using an Accuri C6 flow cytometer and standard settings.

Pre-incubation with LPS is required to obtain secretion of IL-1β by THP-1 cells upon exposure to ABAs or dealuminated (USY), although no significant difference in the exposure of calreticulin and HMGB1 was observed whether the THP-1 cells were pre-incubated with LPS or not. In all experiments examining surface expression or secretion of IL-1β into the medium, THP-1 cells, 1 × 10^6^ cells/ml, were pre-incubated with 1 μg LPS/ml in R10 medium at 37 °C during 4 h. The cells were harvested by centrifugation, re-suspended in R10, co-cultured with ABAs or USY and stained with antibodies against IL-1β, calreticulin or HMGB1. In parallel, as control, THP-1 cells were pre-incubated in R10 during 4 h before harvest and co-culturing with ABAs or USY.

### Pre-labelling of aluminium adjuvants

ABAs, corresponding to 4 mg aluminium per ml, were incubated overnight at room temperature on a rocking table with 50 μM lumogallion in a total volume of 1.0 ml 150 mM NaCl. The next day, the adjuvants were collected by centrifugation for 10 min at 13,000×*g*. The collected adjuvants were re-suspended at 4 mg/ml in 150 mM NaCl and stored in the refrigerator until further use [18, 19].

### Co-culture with pre-labelled aluminium adjuvants

Triplicates of THP-1 cells, 0.5 × 10^6^ cells per ml, were co-cultured overnight at 37 °C in 96-well plates with Alhydrogel or Adju-Phos pre-labelled with lumogallion, at concentrations of 100 μg/ml in a total volume of 200 μl R10. Cells cultured in R10 were used as controls. The next day, triplicates from each incubation were pooled and centrifuged for 5 min at 1000×*g* and re-suspended in PBS containing 0.1% (*w*/*v*) BSA and 0.1% (*w*/*v*) human IgG at 1 × 10^6^ cells per ml. The cells were sub-divided into aliquots and stained with APC-labelled anti-human calreticulin, anti-human HMGB1 or an APC-labelled isotype control by incubation for 30 min on ice. Finally, the cells were washed with PBS containing 0.1% (*w*/*v*) BSA, re-suspended in 250 μl 1% (*w*/*v*) paraformaldehyde (PFA) and analyzed by flow cytometry using an Accuri C6 flow cytometer with standard settings.

### Intracellular staining

THP-1 cells, 1 × 10^6^ cells per ml, were co-cultured with ABAs corresponding to final aluminium concentrations ranging from 25 to 100 μg/ml during 1 to 16 h (overnight) at 37 °C. Controls were cells cultured in R10 in the absence of aluminium adjuvant. The cells were harvested, washed with PBS and divided into two fractions. One cell fraction was re-suspended in PBS containing 0.1% (*w*/*v*) BSA and 0.1% (*w*/*v*) human IgG and surface stained using APC-labelled anti-human HMGB1 or an APC-labelled isotype control. The second fraction was permeabilised using the BD Cytofix/Cytoperm™ Fixation/Permeabilization Kit (BD Bioscience, San Jose, CA, USA) and intracellularly stained using APC-labelled anti-human HMGB1 or an APC-labelled isotype control.

Finally, the cells were analysed by flow cytometry using an Accuri C6 flow cytometer with standard settings.

### Confocal microscopy

THP-1 cells were co-cultured overnight with ABAs pre-labelled with 50 μM lumogallion and stained with APC-conjugated anti-human calreticulin, anti-human HMGB1 or an APC-labelled isotype control, as described earlier. After re-suspension in 1% (*w*/*v*) PFA, the cell suspensions were incubated for 15 min at room temperature. The cells were collected by centrifugation for 5 min at 1000×*g* and washed twice with 500 μl PBS. Finally, the cells were re-suspended in a small volume of PBS and mounted on microscope slides using ProLong® Gold Antifade Mountant with DAPI (Life Technologies, ThermoFisher Scientific, MA USA). After mounting, the samples were analysed on a Zeiss LSM 780 confocal microscope (Carl Zeiss Microscopy GmbH, Jena, Germany). DAPI was excited at 405 nm and the 410–493-nm emission was recorded; lumogallion was excited at 488 nm and the 534–607-nm emission was recorded and APC-labelled antibodies were excited at 633 nm and the 650–743-nm emission was recorded. Z-stack images were obtained at ×63 magnification and analysed with ZEN 2012 (Carl Zeiss Microscopy GmbH).

### Determination of HMGB1 and IL-1β in culture medium

Culture supernatants collected as described in the “Co-culture with aluminium adjuvants” section were thawed, and the content of HMBG1 and IL-1β in the culture medium was assayed using ELISA (HMGB1 ELISA, IBL International GMBH, Hamburg, Germany and DuoSet, Human IL-1β DuoSet ELISA, R&D systems, MN, USA), performed according to the manufacturer’s instructions. The HMGB1 content was assayed using the high sensitive range and 50 μl sample volume. The IL-1β content was assayed using a sample volume of 100 μl.

### Adsorption of HMGB1 and IL-1β by aluminium adjuvants

ABAs, 400 μg/ml, were conditioned by overnight incubation in R10 at 37 °C. The next day, conditioned ABAs were diluted with R10 to 40 and 4 μg/ml. Conditioned ABAs were then incubated overnight at 37 °C in an equal volume of R10 containing HMGB1 or IL-1β. The next day, supernatants from the incubations were harvested by centrifugation for 10 min at 13,000×*g*. The supernatants were stored at − 80 °C until the HMGB1 or IL-1β content was determined by ELISA.

### Isolation of human peripheral monocytes and co-culture with aluminium adjuvants

MACS technology based on magnetic labelling of cells and retaining cells on a column was used to isolate monocytes (Monocyte isolation kit II, Miltenyi Biotec, Bergisch Gladbach, Germany).

Briefly, peripheral blood mononuclear cells (PBMCs) were obtained from buffy coat from healthy donors by density centrifugation on Ficoll-Paque™ (GE Healthcare Life Sciences, Uppsala, Sweden).

Untouched CD14^+^ monocytes were isolated by indirect magnetic labelling of non-monocytes with a cocktail of biotin-conjugated antibodies against CD3, CD7, CD16, CD19, CD56, CD123 and CD235a followed by the addition of anti-Biotin MicroBeads. Non-CD14^+^ monocytes were depleted on a MACS column, and cells in the flow-through were collected, washed and re-suspended in R10 medium at 1 × 10^6^ cells per ml.

Quadruplicates of isolated peripheral monocytes, final concentration 0.5 × 10^6^ cells per ml, were incubated in 96-well plates with ABAs corresponding to final aluminium concentrations ranging from 25 to 100 μg/ml in a total volume of 200 μl R10, during 1 h at 37 °C. Controls consisted of cells cultured in R10 in the absence of ABAs. Cells from the four wells of each incubation were pooled and centrifuged for 5 min at 1000×*g*. Collected cells were re-suspended in PBS containing 0.1% (*w*/*v*) BSA and 0.1% (*w*/*v*) human IgG at 1 × 10^6^ cells per ml. The cells were sub-divided into aliquots and stained by incubation for 30 min on ice with APC-labelled anti-human calreticulin, anti-human HMGB1 or an APC-labelled isotype control. Finally, the cells were washed with PBS containing 0.1% (*w*/*v*) BSA, re-suspended in 250 μl 1% (*w*/*v*) paraformaldehyde (PFA) and analysed by flow cytometry using an Accuri C6 flow cytometer with standard settings.

The remaining cells, not stained with antibodies, were incubated with 7AAD (BD Bioscience, San Jose, CA, USA) for 10 min at room temperature before the viability of the cell preparations was assayed by flow cytometry.

## Results

**Fig. 1:**
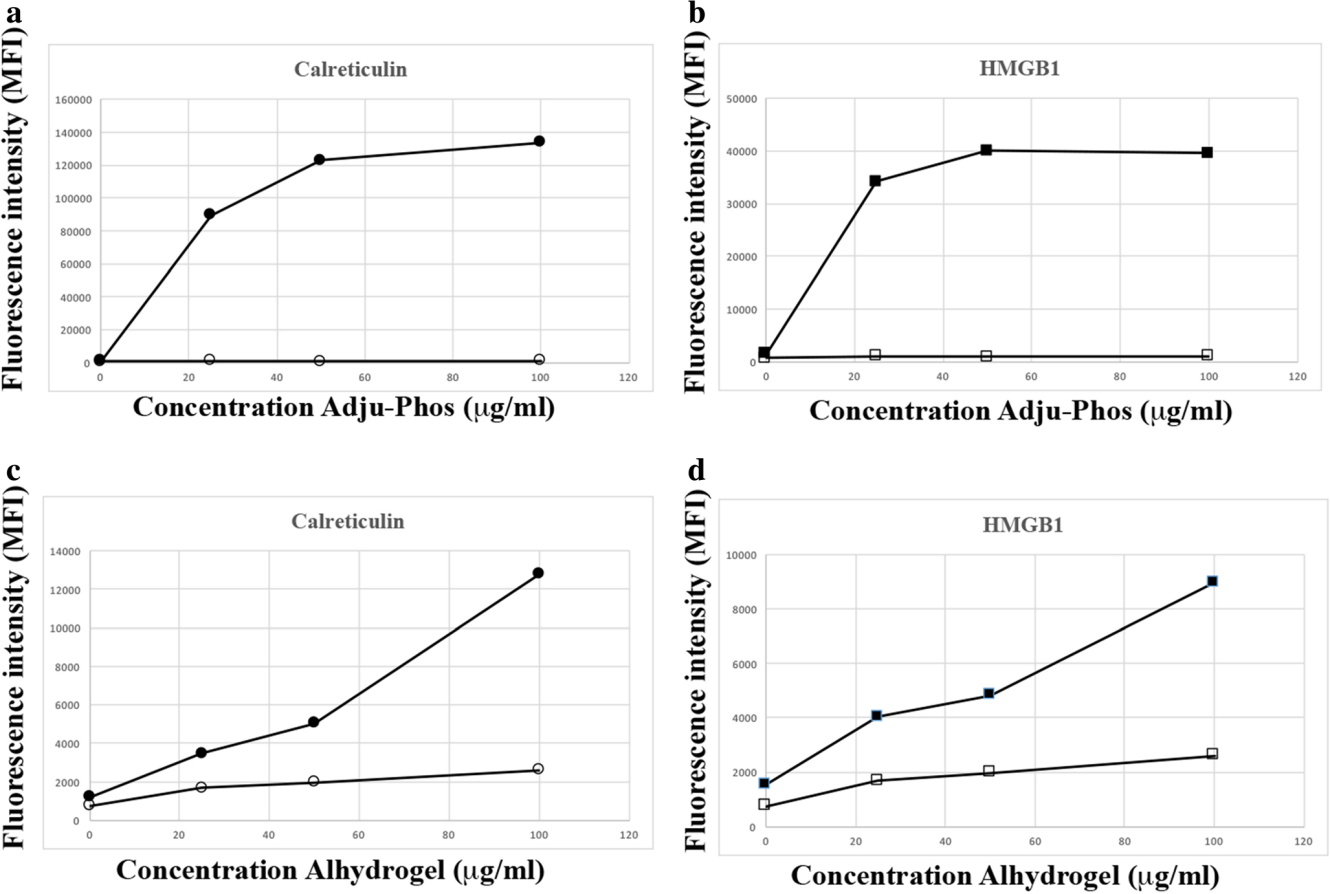
Surface expression of calreticulin and HMGB1 after co-culturing with ABAs. THP-1 cells were co-cultured overnight with various amounts of ABAs and then stained with antibodies against calreticulin, HMGB1 or an isotype control antibody. The cells were analysed by flow cytometry, and the mean channel fluorescence intensity (MFI) from the cells was measured. **a** Cells co-cultured with Adju-Phos and stained with APC-labelled anti-calreticulin (black circle) or isotype control (white circle). **b** Cells co-cultured with Adju-Phos and stained with APC-labelled anti-HMGB1 (black square) or isotype control (white square). **c** Cells co-cultured with Alhydrogel and stained with APC-labelled anti-calreticulin (black circle) or isotype control (white circle). **d** Cells co-cultured with Alhydrogel and stained with APC-labelled anti-HMGB1 (black square) or isotype control (white square)

The DAMPs calreticulin and HMGB1 were expressed on cell surfaces of the human monocytic leukaemia cell line THP-1, after co-culturing with ABAs (Fig. 1). Surface detection of DAMP molecules was done using flow cytometry with a gate setting on viable cells, as determined by less than 1% 7AAD-positive cells in the gate, and no reduced proliferation upon cultivation of the cells during 3 days in the presence of the investigated concentrations of ABAs. A higher expression of both DAMP molecules was observed after co-culturing with Adju-Phos compared to Alhydrogel and this applied especially to calreticulin, in which Adju-Phos showed at least one order of magnitude higher expression.

**Fig. 2:**
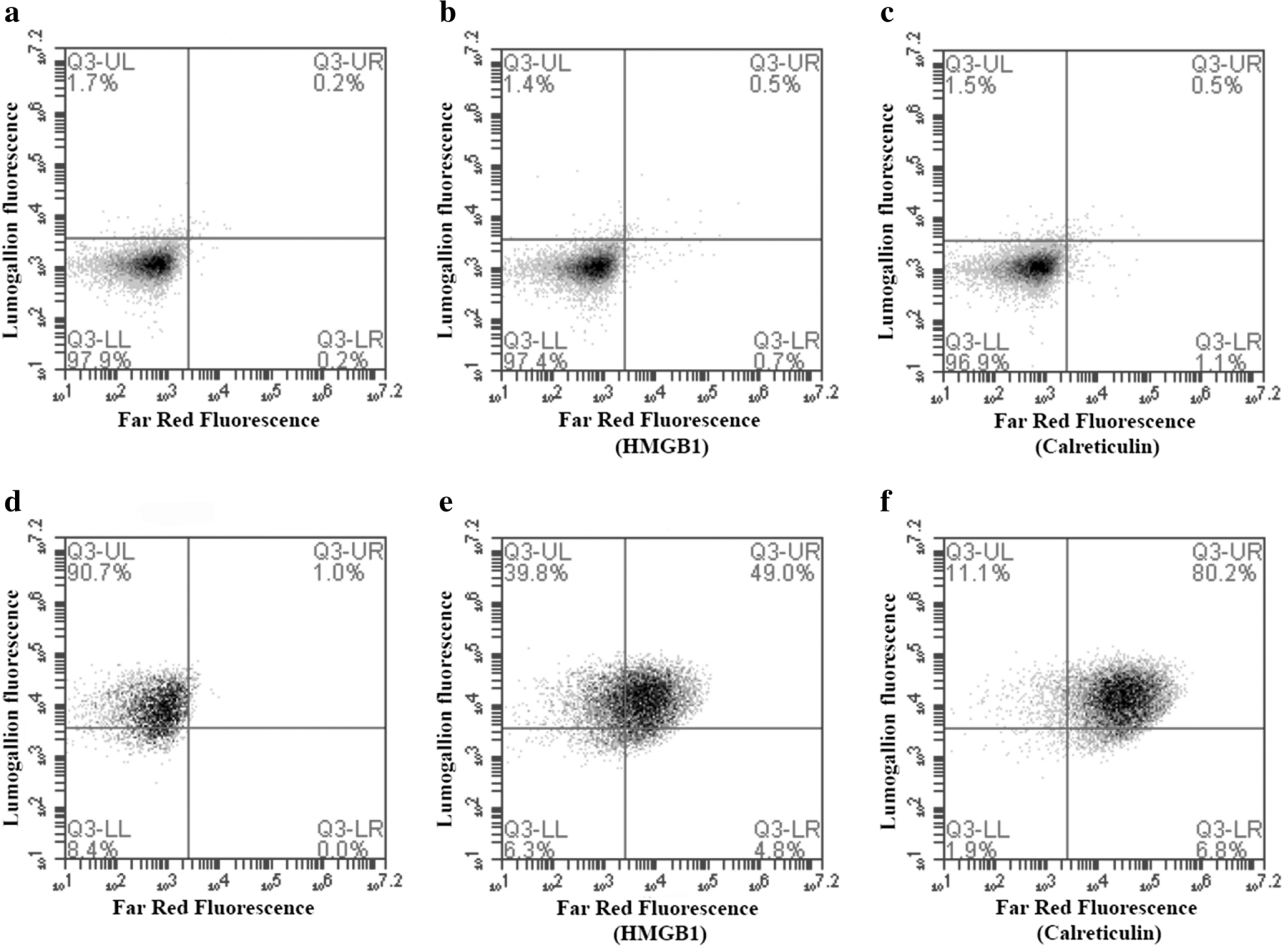
Surface expression of calreticulin and HMGB1 after co-culturing with Adju-Phos. THP-1 cells were co-cultured overnight with lumogallion-labelled Adju-Phos and then stained with APC-labelled antibodies against calreticulin, HMGB1 or an isotype control antibody. The cells were analysed by flow cytometry showing antibody binding (calreticulin or HMGB1 expression) and endocytosed Al adjuvant (lumogallion fluorescence). **a**–**c** THP-1 cultured in R10. **d**–**f** THP-1 co-cultured with 100 μg/ml lumogallion-labelled Adju-Phos. **a**, **d** Cells stained with isotype control antibodies. **b**, **e** Cells stained with anti-HMGB1. **c**, **f** Cells stained with anti-calreticulin

Endocytosed ABAs can be detected by histochemical staining using lumogallion [18] with, however, ABAs can also be pre-stained with lumogallion in the design of fluorescent and traceable ABAs, and their extra- or intracellular location after co-culturing with cells can easily be identified [19]. A clear correlation between DAMP expression and cellular adjuvant association was observed after co-culturing of THP-1 cells with lumogallion pre-labelled Adju-Phos and staining with APC-conjugated antibodies against calreticulin and HMGB1 (Fig. 2).

**Fig. 3:**
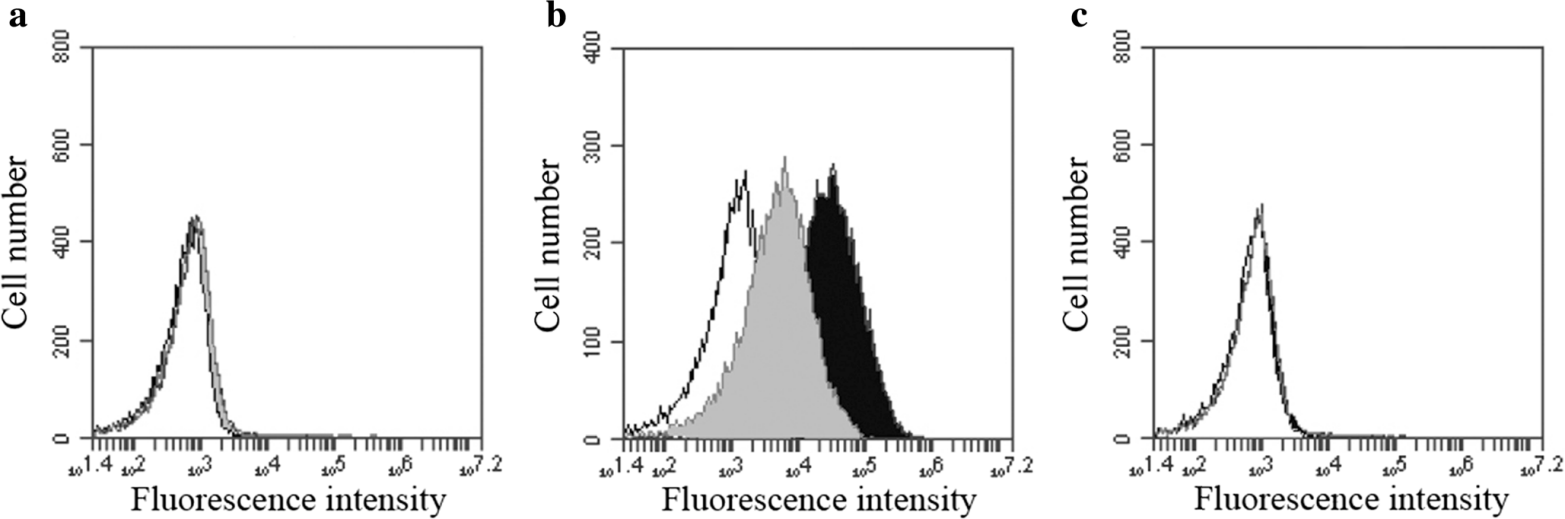
Surface expression of calreticulin and HMGB1 after co-culturing with Adju-Phos or dealuminated zeolite Y. **a** THP-1 cultured in R10. **b** THP-1 co-cultured with 100 μg/ml Adju-Phos. **c** THP-1 co-cultured with 10 μg/ml dealuminated zeolite Y (USY). Cells stained with isotype control antibody (white histogram), cells stained with anti-HMGB1 (grey histogram) and cells stained with anti-calreticulin (black histogram)

Dealuminated zeolite Y (USY) is almost insoluble at neutral and slightly acidic pH and USY particles of an average size of 1.2 μm, determined by dynamic light scattering, which is approximately the same size as the ABAs, 1.4 μm [20], were used to certify that DAMP expression was not merely induced by the internalization of non-degradable particles in the endosomal pathway. In control experiments, no surface expression of calreticulin or HMBG1 was observed after co-culturing cells with USY, at zeolite concentrations just below the toxicity and growth inhibition threshold (Fig. 3).

**Fig. 4:**
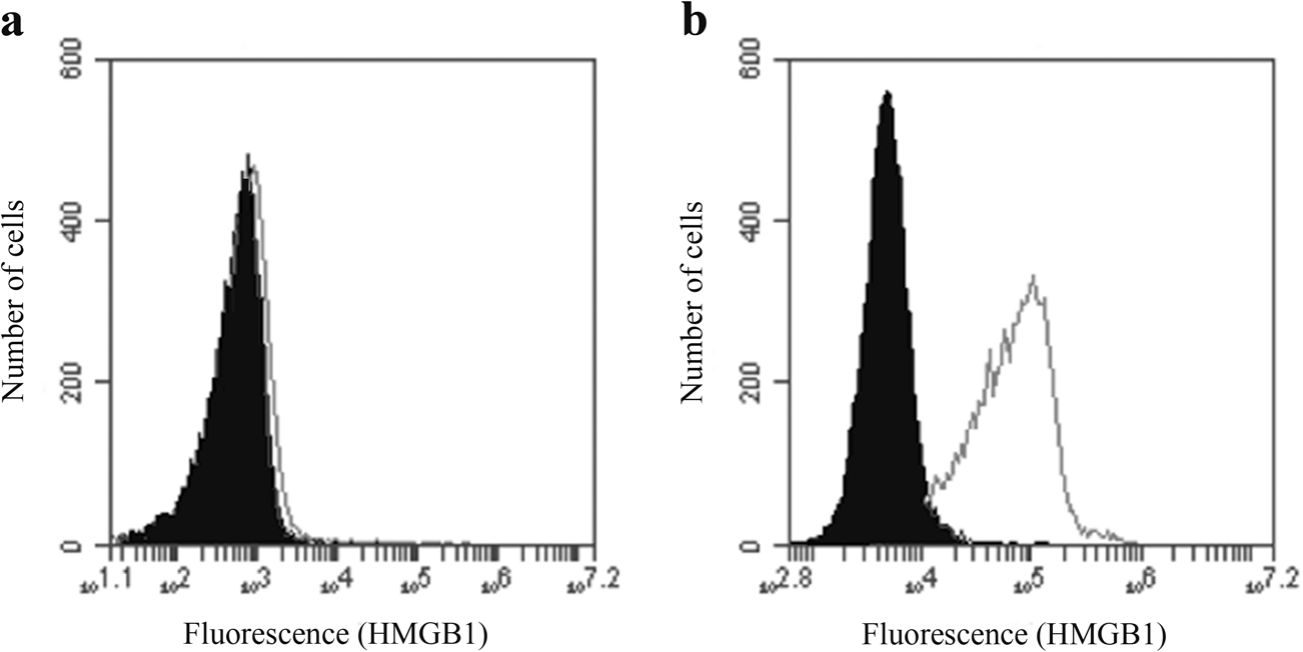
Intracellular HMGB1 staining of THP-1 cells. **a** Surface staining of THP-1 cells. **b** Staining of permeabilised cells. Cells stained with an isotype control antibody (black histogram) and cells stained with anti-HMGB1 (white histogram)

THP-1 cells have a high content of intracellular HMGB1 as shown by intracellular staining of cells cultured in medium without added ABA (Fig. 4). However, no significant reduction of intracellular HMGB1 was observed after permeabilization and staining of cells co-cultured with adjuvants.

**Fig. 5:**
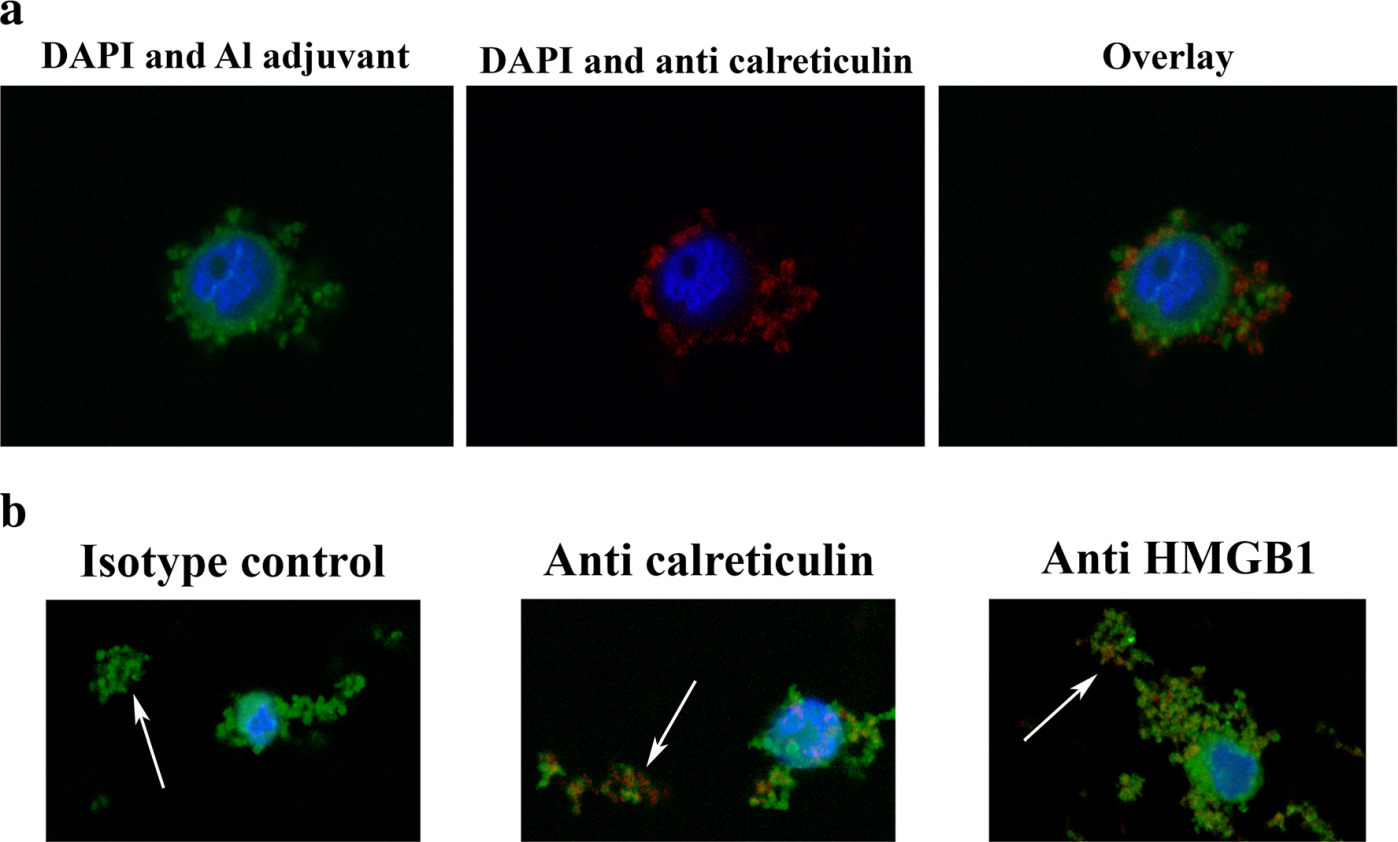
**a** Confocal images of THP-1 cells co-cultured with lumogallion-labelled Adju-Phos and stained with anti-calreticulin. THP-1 cells were co-cultured over night with 200 μg/ml lumogallion-labelled AdjuPhos. The next day, the cells were stained with APC-labelled anti-calreticulin, washed with PBS, fixed in PFA and mounted using ProLong® Gold Antifade Reagent. A confocal z-stack was made and the centre of a cell is shown in the figure. To the left: nuclear (blue, DAPI) and Adju-Phos staining (green, lumogallion). In the middle: nuclear (blue, DAPI) and anti-calreticulin staining (red, APC). To the right: an overlay image showing nucleus (blue), Adju-Phos adjuvant (green) and anti-calreticulin (red) staining. **b** Confocal images of aggregates of lumogallion-labelled Adju-Phos stained with anti-HMGB1 and anti-calreticulin. Cells were co-cultured with lumogallion-labelled Adju-Phos as described in Fig. 3a. After co-culturing, the cells were stained with APC-labelled antibodies against calreticulin, HMGB1 or an isotype control antibody; washed with PBS; fixed in PFA and mounted using ProLong® Gold Antifade Reagent. Confocal z-stacks were made, and a section slightly below the cell centre showing a cell and non-endocytosed Adju-Phos aggregates is shown in the figure. Arrows pointing at non-endocytosed Adju-Phos aggregates. To the left: an overlay image after staining with the isotype control antibody. In the middle: an overlay image after staining with anti-calreticulin. To the right: an overlay image after staining with anti-HMGB1. Nucleus stained with DAPI (blue), Adju-Phos adjuvant stained with lumogallion (green) and antibodies labelled with APC (red)

Although THP-1 cells showed a high surface expression of HMGB1, no HMGB1 could be detected by ELISA in the medium of THP-1 cells co-cultured with ABAs (data not shown). Antibodies cannot penetrate an intact cell membrane, and by confocal microscopy of non-permeabilised cell, both intracellular cell membrane structures can be evaluated. Confocal microscopy of cells co-cultured with lumogallion-labelled ABAs confirmed surface staining of antibodies against calreticulin and an intracellular presence of ABAs. (Fig. 5a). However, overlay images of antibody and adjuvant staining also showed co-localization of antibodies and ABAs on the cell surface, implying a cell surface association between DAMP molecules and ABA (Fig. 5a).

The samples in the confocal microscopy studies also held extracellular, i.e. non-endocytosed, ABA particles, and by adjusting the focal depth, antibodies against calreticulin and HMGB1 bound to the extracellular ABA particles were observed (Fig. 5b).

**Fig. 6:**
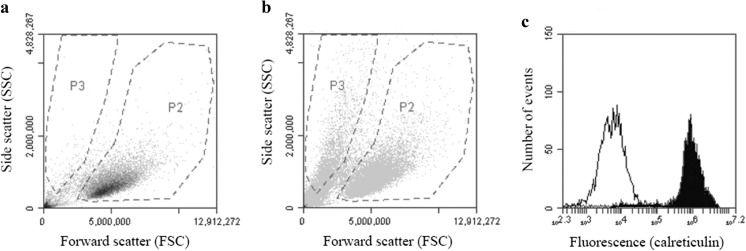
Gate setting and detection of antibody binding to the cell surface and to extracellular aluminium adjuvant particles. THP-1 cells were co-cultured overnight with or without Adju-Phos, stained with APC conjugated antibodies and analysed by flow cytometry with the same number of events collected in the cell gate, P2. **a** Forward and side scatter dot plot of THP-1 cultured in R10 and stained with an isotype control antibody. **b** Forward and side scatter dot plot of THP-1 co-cultured with Adju-Phos, 100 μg/ml, and stained with an isotype control antibody. **c** Fluorescence histogram of events detected in the P3 gate, as shown in the dot plot, of THP-1 cells co-cultured with Adju-Phos, 100 μg/ml, and stained with isotype control antibody (white histogram) or anti-calreticulin (black histogram)

In a standard flow cytometry experiment, the same number of cells is collected in each experiment regardless of the volume, i.e. in all samples, the same number of cell-sized events is collected in a cell gate as shown in Fig. 6a, b, gate P2. This enables the same statistical sampling of each sample regardless of the presence of debris or in this case, debris and ABA particles not associated with the cells in the samples. Debris is visualised as events close to the origo of the dot plot, and by making a particle gate not too close to origo, shown as gate P3 in Fig. 6a, b, a fraction of the non-endocytosed ABA particles in the samples could be analysed. Collecting the same number of cells in all samples resulted in less than 200 events (or debris) in the particle gate, P3, after co-culturing with R10 alone, whereas more than 2500 events were collected after co-culturing in the presence of 100 μg ABA/ml. Thus, a gate setting as outlined in Fig. 6a, b made it possible to discriminate between cells and extracellular aluminium adjuvant particles, thereby enabling the detection of antibodies bound to extracellular ABAs, i.e. DAMP molecules bound to extracellular ABA after co-culturing with THP-1 (Fig. 6c).

**Fig. 7:**
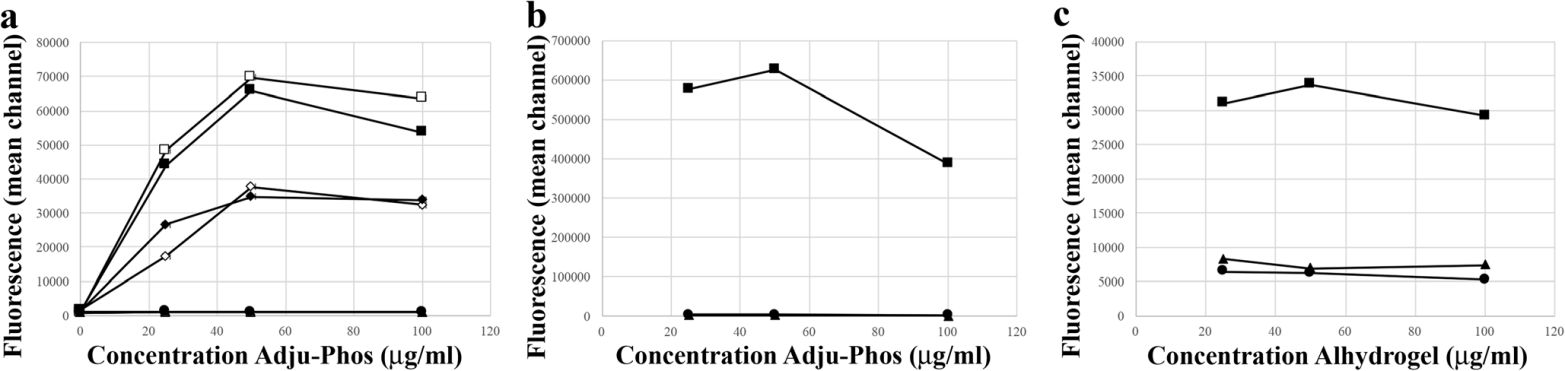
Staining of THP-1 cells pre-stimulated with LPS. THP-1 cells were either native (open symbols) or pre-stimulated with LPS (filled symbols), and then co-cultured with various concentrations of ABAs. **a** Cell surface expression of calreticulin, HMGB1 and IL-1β. THP-1 cells co-cultured with Adju-Phos, stained with antibodies and analysed by flow cytometry, collecting cell events in the P2 gate as shown in Fig. 6. Cells stained with APC-labelled anti-calreticulin (black square, white square), anti-HMGB1 (black diamond, white diamond), anti-IL-1β (black triangle, white triangle) or an isotype control (black circle, white circle). Data presented as averages of four independent experiments. **b** Presence of calreticulin and IL-1β on extracellular Adju-Phos particles. THP-1 cells pre-stimulated with LPS were co-cultured with various concentrations of Adju-Phos, stained with antibodies and analysed by flow cytometry, collecting extracellular adjuvant particles in the P3 gate as shown in Fig. 6. Co-culture stained with APC-labelled anti-calreticulin (black square), anti-IL-1β (black triangle) or an isotype control (black circle). Data presented as averages of four independent experiments. **c** Presence of calreticulin and IL-1β on extracellular Alhydrogel particles. THP-1 cells pre-stimulated with LPS were co-cultured with various concentrations of Alhydrogel, stained with antibodies and analysed by flow cytometry, collecting extracellular adjuvant particles in the P3 gate as shown in Fig. 6. Co-culture stained with APC-labelled anti-calreticulin (black square), anti-IL-1β (black triangle) or an isotype control (black circle). Data presented as averages of four independent experiments

Pre-stimulation with LPS enables the secretion of IL-1β into the medium after co-culturing with ABAs [9, 21] or USY, and a dose-dependent amount of IL-1β, ranging from 50 to 250 pg/ml, was achieved upon co-culturing with ABAs or USY. THP-1 cells showed no significantly different surface exposure of calreticulin or HMGB1 upon pre-incubation with LPS (Fig. 7a), and no specific binding of anti-IL-1β compared to isotype control was observed at the cell surface (Fig. 7a). After co-culturing with THP-1 cells, using a gate setting on extracellular ABA particles, see gate P3 in Fig. 6, revealed antibody binding to the particles and indirectly the presence of DAMPs or IL-1β adsorbed onto the ABA particles. Anti-calreticulin showed a dose-dependent binding onto Adju-Phos particles, whereas in comparison, Alhydrogel particles showed a weaker and an almost constant binding of anti-calreticulin (Fig. 7b, c). A similar, although a much weaker, binding was obtained after staining with anti-HMGB1. However, no significant binding of anti-IL1β onto the extracellular particles was observed whether the cells had been pre-incubated with LPS or not, although IL-1β was present in the medium after co-culturing with THP-1 cells pre-incubated with LPS; no adsorption of IL-1β was observed onto the ABA particles (Fig. 7b, c).

To further investigate the adsorption of DAMP molecules onto ABAs, medium containing HMGB1 or IL-1β was incubated with ABAs pre-incubated with culture medium supplemented with 10% foetal calf serum. The pre-incubation step of the ABAs was done to ensure total protein adsorption onto the ABAs and thereby blocking the antigen-binding capacity before exposure to HMGB1 or IL-1β. Both Alhydrogel and Adju-Phos completely depleted the medium of HMGB1 at adjuvant concentrations used in the co-culture experiments with THP-1 cells. However, at lower adjuvant concentrations, Alhydrogel had a higher adsorption capacity compared to Adju-Phos (Table 1). IL-1β on the other hand showed no adsorption onto the ABAs, and no depletion of IL-1β took place upon incubation with either Alhydrogel or Adju-Phos.

**Table 1:** Adsorption of HMGB1 by aluminium adjuvant particles

Adjuvant (μg/ml)	HMGB1 in solution (ng/ml)		HMGB1/Al adjuvant (ng/μg)	
	Alhydrogel	Adju-Phos	Alhydrogel	Adju-Phos
0	14.1	14.1	NA^a^	NA^a^
2	3.0	10.4	5.6	1.9
20	0.0	5.3	0.7	0.4
200	0.0	1.8	0.07	0.06

Alhydrogel and Adju-Phos were pre-incubated with R10 overnight at 37 °C. The next day, the adjuvants were diluted with R10 to 400, 40 and 4 μg/ml and mixed with an equal volume of R10 containing HMGB1. The samples were then incubated overnight at 37 °C again. After incubation, the adjuvant particles were removed by centrifugation and the content of HMGB1 in the supernatants was determined by a HMGB1-ELISA

^a^NA not applicable

**Fig. 8:**
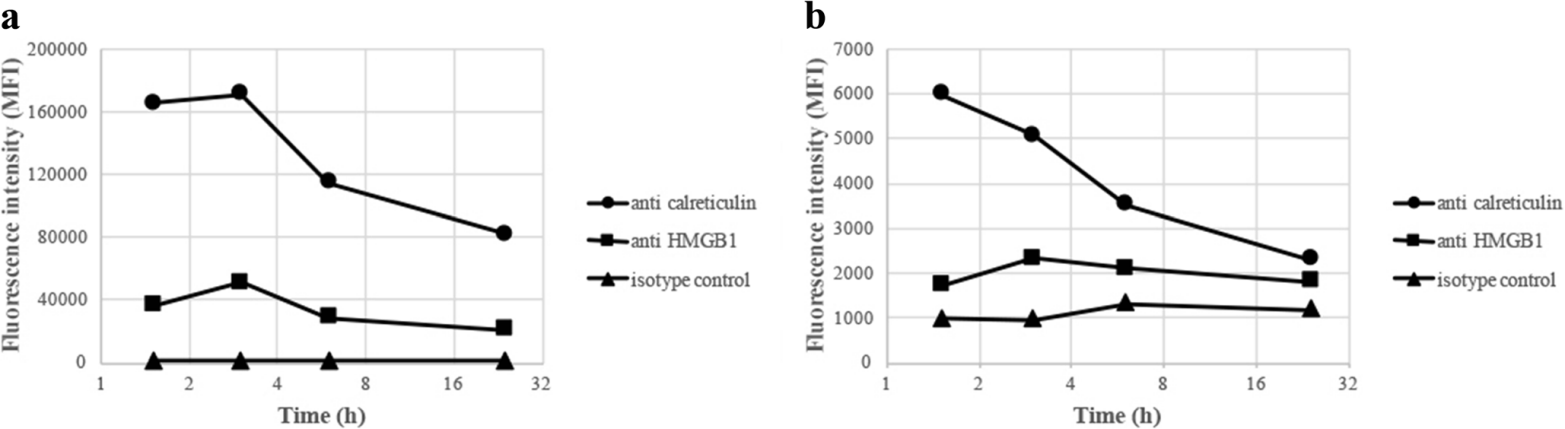
Kinetic surface expression of calreticulin and HMGB1 after co-culturing of THP-1 cells and ABAs. THP-1 cells were co-cultured with 50 μg/ml Adju-Phos (**a**) or Alhydrogel (**b**) for various times before the cells were stained with APC labeled anti-calreticulin (black circle), anti-HMGB1 (black square) or an isotype control (black triangle). The cells were analysed by flow cytometry, and the mean channel fluorescence intensity (MFI) from the cells was measured

Compared to overnight incubations, reducing the exposure time between cells and adjuvants increased the surface expression of both calreticulin and HMGB1. Already after 1 to 2 h of co-culture, the most abundant surface expression was obtained (Fig. 8).

**Fig. 9:**
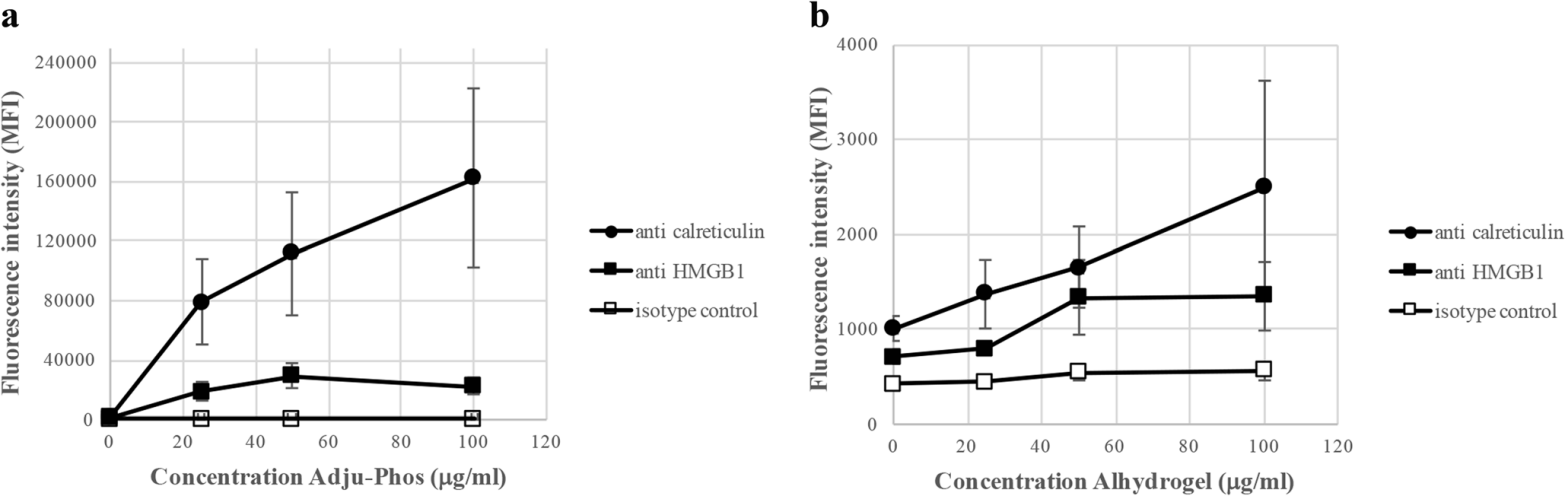
Surface expression of calreticulin and HMGB1 by peripheral monocytes after co-culturing with ABAs. Human peripheral monocytes were co-cultured with various concentrations of ABAs. After 1 h, the cells were stained with APC labelled anti-calreticulin (black circle), anti-HMGB1 (black square) or an isotype control (white square). The cells were analysed by flow cytometry, and the mean channel fluorescence intensity (MFI) from the cells was measured. Data presented as means ± SD from four different donors and independent experiments. **a** Co-culture with Adju-Phos. **b** Co-culture with Alhydrogel

THP-1 cells are commonly used as a model of human monocytes/macrophages, and co-culture of human peripheral monocytes and ABAs resulted in a surface expression of calreticulin and HMGB1 similar to THP-1 cells (Fig. 9). Peripheral monocytes reproduced the THP-1 behaviour, and expression of calreticulin and HMGB1 by peripheral monocytes was at least one order of magnitude higher after co-culturing with Adju-Phos, compared to Alhydrogel.

## Discussion

A supported model regarding sterile inflammation [22] draws on the release of DAMP molecules from damaged, dying or dead cells. DAMP molecules can also be released upon cellular stress [23, 24], and cellular stress is expected after endocytosis of non-degradable particles such as ABAs. In THP-1 cells, a human cell line of monocytic origin, an unfailing surface expression of DAMP molecules could be linked to co-culturing with ABAs (Fig. 1). Co-culturing with the specific amounts of ABAs used did not reduce cellular proliferation, and in all experiments, less than 1% dead cells were included in the assays, indicating that ABAs induced release and expression of DAMP molecules by stressed, yet viable, cells. DAMP molecules are known to provoke inflammation, and an induced pattern of DAMP molecules at the inoculation site of a vaccine will most certainly influence the local inflammation, and ultimately the immunological response. In this report, most of the results were obtained using the monocytic cell line THP-1, but human peripheral monocytes also release and express DAMP molecules upon exposure to ABAs (Fig. 9), suggesting that DAMP induction and presentation by ABAs by viable phagocytic cells is a generalizable feature. This implies that the inflammatory mechanism of ABAs at the inoculation site in vivo needs to be reconsidered.

Intracellular staining revealed high amounts of calreticulin and HMGB1 and even if high surface expression was obtained after co-culturing with ABAs, no reduction of the intracellular content could be observed. Not even Adju-Phos, which induced a considerable surface expression of calreticulin, did significantly reduce the intracellular content, indicating that the adjuvant-induced secretion of DAMPs only involved a minor fraction of the total cellular content.

Confocal microscopy of non-permeabilised cells revealed DAMP molecules associated with ABAs on cell surfaces and with adjuvant particles in the medium not associated with any cells (Fig. 5). Adsorption onto adjuvant particles explains that HMGB1 was not detected in the medium after co-culturing with ABAs (Table 1). Both Alhydrogel and Adju-Phos did adsorb HMGB1 in conditioned culture medium, and Alhydrogel was the most efficient adsorber of the two. It should also be emphasised that IL-1β, one of the few inflammatory mediators that has been reported to be secreted by cells upon endocytosis of ABAs, was not adsorbed by the adjuvant particles.

Adsorption of DAMP molecules onto adjuvant particles was apparent from flow cytometry, using a gate setting highlighting free particles not associated with cells. With a gate setting as outlined in Fig. 6, a fraction containing relatively large ABA particles was detected and the number of events detected in the gate decreased as the ABA concentration in the co-culture was decreased. Cellular debris was to some extent detected in the particle gate, and co-culture of cells at ABA concentrations of 25 μg/ml increased the events in the particle gate 3 to 4 times compared to the number of events that was obtained from cellular debris. Owing to a large bias anticipated by the presence of cellular debris, no attempts were made trying to analyse adsorption of calreticulin and HMGB1 onto ABA particles after co-culturing with lower concentrations of ABAs than 25 μg/ml. However, flow cytometry clearly demonstrated that DAMP molecules were more strongly associated with Adju-Phos particles, whereas the association to Alhydrogel was less pronounced and an almost constant adsorption of DAMP molecules, regardless of adjuvant concentration in the medium, was observed (Fig. 7). Detection of DAMP molecules relies on recognition by antibodies and depending on the adsorption mechanism, epitopes may be hidden or destroyed, explaining the discrepancy between the results obtained by indirect (ELISA of HMGB1 in the medium) and direct (flow cytometry) measurements of DAMP molecules adsorbed onto adjuvant particles.

Adsorption of DAMP molecules onto adjuvant particles appears to be facilitated by some form of specificity, rather than a general protein adsorption mechanism. Specificity is indicated by the fact that both calreticulin and HMGB1 were adsorbed by the ABAs whereas no adsorption of IL-1β was observed during co-culture of cells pre-treated with LPS (Fig. 7).

Adsorption of DAMP molecules or other inflammatory mediators onto adjuvant particles might affect the inflammatory signal. Induction of inflammatory signals by ABAs upon endocytosis and sequential adsorption of some of the secreted alarmins may quench, attenuate or bias the inflammatory process and thereby affect the immune stimulating response. Adsorption of inflammatory mediators may also act as a local depot of inflammatory signals modulating the inflammatory and immune stimulating events, and further in vitro and in vivo studies are needed to address this subject.

The in vitro expression of DAMP molecules was obtained at adjuvant concentrations less than 50 μg ABAs/ml, whereas the typical ABA load in vaccine formulations [25] results in an expected adjuvant concentration at the inoculation site that is one order of magnitude higher than the in vitro concentrations triggering DAMP induction and sequential adsorption. Vaccine development using ABAs has generally been focused on loading the adjuvant with antigens, thus realising a proper dosage of the vaccine based upon a high degree of antigen adsorption onto the ABAs even though reports have detailed the immune stimulating properties of ABAs without any adsorbed antigen and thereby no long-term release of antigen [26, 27]. ABAs are regarded as adjuvants inducing a humoral immune response mainly, and reports have shown that low doses of ABAs do not provide an optimal immune stimulation [28]. In this case, the read-out, measuring the adjuvant effect has been antibody production, and an interesting observation is that optimal antibody production seems to depend on the presence of adjuvant particles not completely adsorbed with antigen and thereby exposing free adsorption sites [29]. It can be speculated whether this reflects immune activation facilitated by the adsorption and attenuation of alarmins or other immune mediators by the ABAs, directing the immune response against a Th2 response and antibody production. Today, the presence of low-abundance antigen-specific T-lymphocytes can be detected and analysed by methodologies such as MHC multimers [30, 31], and analysing antigen-specific T-lymphocytes upon immunization using low and high concentrations of ABAs would be of considerable interest.

Calreticulin is secreted into the surrounding medium and also exposed on the cell surface [14], whereas HMGB1 is believed to be secreted into the medium only [32, 33]. Both calreticulin and HMGB1 were detected on the cell surface after culturing in the presence of ABAs, and cell surface exposure of DAMP molecules is probably mediated through ABAs adsorbed on the surface of the cells. The immune stimulating properties of ABAs have earlier been reported to be mediated through adjuvant interactions with lipid rafts on the cell surface [34], and it can be speculated whether a DAMP—adjuvant facilitated cell—cell interaction and thereby DAMP signalling is involved in this process.

Our results clearly show that Adju-Phos sequesters a much larger number of DAMP molecules on the surface of both THP cells and freshly prepared human monocytes than does Alhydrogel, even though the latter binds free HMBG1 more efficiently. No previous comparisons of these aspects have been made although it has been shown that nanoparticles of either adjuvant induce more Il-1β than the corresponding microparticles [35]. Interestingly, also calcium phosphate has adjuvant properties and has been used in clinical vaccines [36]. Furthermore, chemical modification of adjuvants, e.g. Allhydrogel and Imject, has been reported to manage the immune stimulatory capacity [37]. The implications of the differences between the two adjuvants observed in the present study await validation in relevant in vivo studies.

The immune-stimulating properties of ABAs are believed to be promoted by the onset of an inflammatory process, but relatively few pro-inflammatory mediators induced by ABAs have been reported, including mainly cytokines of the IL-1 family [3, 38]. It can be speculated that the sparsely reported variety of inflammatory mediator upon exposure to ABAs is due to adsorption by the ABAs, making the mediators non-detectable in the medium by ELISA or other kind of assay.

The involvement of DAMP molecules in the immune-stimulating properties of ABAs is of potential interest. An immunological memory of the innate immune system relying on DAMP molecules and epigenetic reprogramming has been hypothesised [39], and if immunization/vaccination would result in an epigenetic reprogramming of the innate immune system [40], induced expression of DAMP molecules by ABAs is most certainly an important aspect. Further investigations will shed light on our understanding of the immune-stimulating properties of ABAs and increase our appreciation of the immune system.
